# Supplementary material for: Six-Minute Walk Distance in Breast Cancer Survivors—A Systematic Review with Meta-Analysis
Source: Int J Environ Res Public Health. 2021 Mar 5;18(5):2591. doi: 10.3390/ijerph18052591 (PMC7967367; doi:10.3390/ijerph18052591)
Supplement: Supplementary file 1 [file ijerph-18-02591-s001.pdf]

Supplementary Table S1. PEDro scores of included studies.

**Author**	**Random allocation**	**Concealed allocation**	**Groups similar at baseline**	**Participant blinding**	**Therapist blinding**	**Assessor blinding**	**<15% dropout**	**Intention to treat analysis**	**Between group difference reported**	**Point estimate and variability reported**	**PEDro score**
Yee	Yes	Yes	Yes	No	No	No	Yes	No	Yes	Yes	6
Hojan	Yes	Yes	Yes	No	No	No	No	No	Yes	No	4
Ariza-Garcia	Yes	Yes	Yes	No	No	No	No	No	Yes	Yes	5
Eyigor	Yes	Yes	Yes	No	No	No	No	No	Yes	No	4
Yuen	Yes	Yes	Yes	No	No	No	No	No	Yes	Yes	5
Vardar-Yagli	Yes	Yes	Yes	No	No	No	No	No	Yes	No	4
Milecki	Yes	No	Yes	No	No	No	No	No	Yes	No	3
Cornette	Yes	Yes	Yes	No	No	No	No	Yes	Yes	No	5
